# Correlations Between Single-Photon Emission Computed Tomography Parameters of Salivary Glands and Dry Eye Tests in Patients With Sjögren's Syndrome

**DOI:** 10.3389/fmed.2021.781382

**Published:** 2022-01-14

**Authors:** Xiaoyu Huang, Lingjuan Xu, Wei Wang, Weikun Hu, Xinyu Li, Hong Zhang, Jing Chen, Guigang Li

**Affiliations:** ^1^Department of Ophthalmology, Tongji Medical College, Tongji Hospital, Huazhong University of Science and Technology, Wuhan, China; ^2^Department of Nuclear Medicine, Tongji Medical College, Tongji Hospital, Huazhong University of Science and Technology, Wuhan, China

**Keywords:** dry eye, Sjögren's syndrome, salivary glands, single photon emission computed tomography (SPECT), Schirmer's I test

## Abstract

**Objective:**

To evaluate the correlations between Single-Photon Emission Computed Tomography (SPECT) parameters of salivary glands and dry eye parameters in patients with Sjögren's syndrome (SS).

**Methods:**

A total of 28 patients with SS participated in this prospective study. Dry eye assessments include tear film break-up time (TBUT), corneal fluorescein staining scoring (CFS), Schirmer's I test (SIT) examination and SPECT of salivary gland. The following quantitative parameters were derived from SPECT imaging for salivary glands: Uptake index (UI), the time needed to achieve the minimum counts after Vit C stimulation (T_s_), and excretion fraction (EF). The relation between the aforementioned parameters and TBUT, CFS and SIT were analyzed with SPSS 22.0 software.

**Results:**

All the 28 eyes of the 28 subjects were examined. The mean SIT was 6.04 ± 4.64 mm/5 min (0–18 mm/5 min); the mean CFS was 3.07 ± 2.65 (0–10) and the mean BUT was 2.11 ± 1.97 s (0–9 s). The mean EF value was 0.52 ± 0.12 (0.26–0.75) in parotid glands and 0.45 ± 0.10 (0.30–0.67) in submandibular glands, respectively. The mean UI value was 9.33 ± 1.68 (6.03–13.20) in parotid glands and 9.92 ± 1.48 (7.08–12.60) in submandibular glands, respectively. The mean T_s_ (min) was 5.32 ± 3.01 (2.00–12.00) in parotid glands and 11.09 ± 7.40 (2.00- 29.00 min) in submandibular glands, respectively. It was found that EF positively correlates with SIT in patients with SS (r = 0.499 and 0.426 in parotid glands and submandibular glands, with *P* < 0.05), while no significant correlation was found between the UI, T_s_ and CFS, TBUT (*P* > 0.05).

**Conclusions:**

The EF was positively correlated with SIT in patients with SS, it could reflex the dysfunction of salivary glands in SS patients. So, EF may be a valuable parameter for the diagnosis of SS patients with lacrimal gland secretion dysfunction.

## Introduction

Sjögren's syndrome (SS) is a chronic autoimmune disorder that typically affects the exocrine glands (mainly salivary and lacrimal glands). Patients who suffer from this condition experience dry mouth and also very dry eyes. The estimated prevalence of this medical condition varies between 0.5 and 1% of the general population (1). Dry eye disease (DED) and dry mouth are typical symptoms of SS. It's reported that 11.6% of patients with clinically significant aqueous-deficient dry eye (ADDE) had SS in an prospective, industry-sponsored, multicentre clinical trial (2).

According to the SS diagnostic criteria of 2016 American College of Rheumatology/European League Against Rheumatism (ACR-EULAR) (3), ocular and oral problems are the main symptoms of SS patients. Ocular examination mainly includes the symptom of ocular dryness, ocular staining score and Schirmer's test (3). Gland biopsy is a method that can provide directly evidence of glandular inflammation, but it is invasive and cause discomfort to the patient, which make it challenging to get early diagnosis.

Single-Photon Emission Computed Tomography (SPECT) can detect nuclide radioactivity and generate images of the human body at both planar and three-dimensional levels. This type of scan has been suggested as a sensitive, dynamic, and objective alternative approach to functional and morphological evaluation of salivary glands of SS patients (4–7). However, the role of SPECT in the diagnosis of SS has been weakened (3, 8), mainly because of the lack of specific criteria for quantitative evaluation of salivary gland SPECT in patients under these conditions (9). The aim of this study is to evaluate the correlations between SPECT parameters of salivary glands and dry eye tests in patients with Sjögren's syndrome.

## Methods

The procedures conducted for study are consistent with the Helsinki Declaration and approved by the Ethics Committee of Tongji Hospital. All subjects have been informed of the aim of study, the principles of related examination methods and possible adverse consequences. Each individual signed an informed consent under totally comprehension.

### Research Objects

Twenty eight patients with SS were enrolled in the study, consulting in the department of Ophthalmology, Rheumatology and Immunology at Tongji Hospital of Tongji Medical College, Huazhong University of Science & Technology (HUST).

Inclusion criteria:

1. 18 to 65 years old.
2. According to the 2016 ACR-EULAR (3), the criteria are based on five objective tests/items: labial salivary gland with focal lymphocytic sialadenitis and focus score of ≥1 foci/4 mm^2^ (3 points), anti-SSA/Ro-positive (3 points), ocular staining score ≥5 (or van Bijsterveld score ≥4) in at least one eye (1 point), Schirmer's test ≤5 mm/5 min in at least one eye (1 point), unstimulated whole saliva flow rate ≤0.1 mL/min (1 point).

Individuals are classified as having primary SS if they have a total score of ≥4.

Exclusion criteria:

1. 3 months before the screening visit with ocular trauma, inflammation of the eyelid infection.
2. Contact lens wearer.
3. Severe heart, lung, liver, kidney dysfunction.
4. Pregnant and lactating women. Psychopaths.

### Research Methods

#### SPECT Examination of Salivary Glands

SPECT was performed with a dual-head Discovery NM/CT 670 SPECT/CT instrument (General Electric Medical Systems, Waukesha, WI, USA) fitted with a low-energy, general-purpose, parallel-hole collimator with standard peak energy settings (20% at 140 keV). The subject remained in supine position, and the probe was positioned for an anterior head-and-neck projection. Dynamic images were immediately acquired in a 128 × 128-pixel matrix, at 1 min per frame. The process lasted for 30 min after a bolus intravenous injection of 370 MBq of 99 mTc-sodium pertechnetate. Fifteen minutes after the injection, each subject was administered 300 mg VitC (sublingual application) without moving, while imaging was continued.

**Figure 1:**
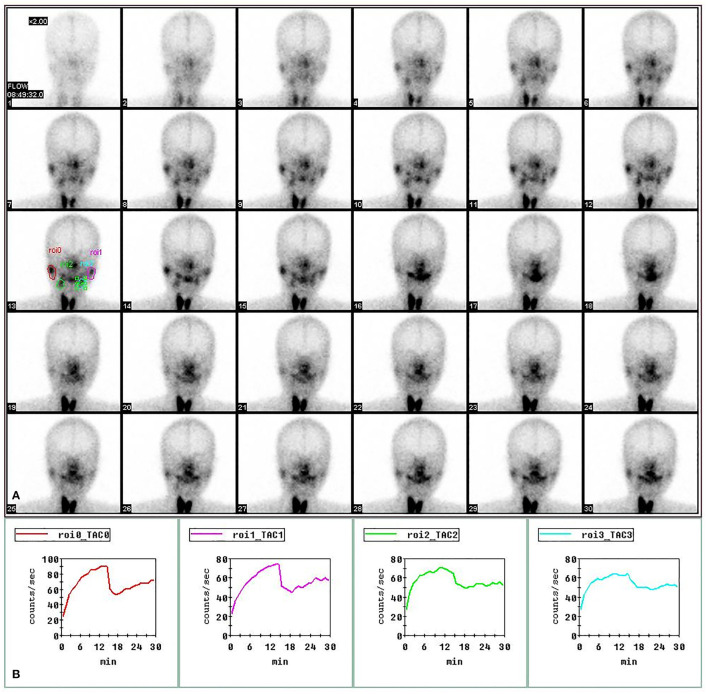
Regions of interest (ROIs) in SPECT imaging of salivary gland. The images of bilateral parotid and submandibular glands at 1–30 min post injection of 370 MBq of 99 mTc-sodium pertechnetate in the upper panel **(A)**. The time-activity curves for right parotid gland (red), left parotid gland (purple), right submandibular gland (green) and left submandibular gland (blue) in the lower panel **(B)**.

Regions of interests (ROI) were drawn on the compressed dynamic images of the parotid and submandibular glands for both right and left sides. Time activity curves were generated for each gland (Figure 1). The following quantitative parameters were derived by nuclear medicine physicians from the department of Nuclear Medicine, Tongji Hospital, Tongji Medical College, HUST. Then, the mean values of each pair of glandular parameters were analyzed.

C_p_ = maximum counts before VitC stimulation.
C_0_ = counts at 1st min.
C_v_ = minimum counts after VitC stimulation.
C_b_ = background count, obtained from the average count of bilateral frontal regions.
Uptake index (UI) = (C_p_-C_0_)/C_0_.
The time after stimulation (T_s_) = time needed to achieve the minimum counts after VitC stimulation.
Excretion fraction (EF) = (C_p_-C_v_)/(C_p_-C_b_).

#### Dry Eye Assessments

The right eyes of all patients were included in the study. All enrolled patients were diagnosed with DED according to the Tear Film and Ocular Surface Society (TFOS) Dry Eye Workshop (DEWS) II (10). The dry eye assessments were performed in the order of tear film break-up time (TBUT), corneal fluorescein staining scoring (CFS), Schirmer's I test (SIT).

#### TBUT

The examiners pulled down the eyelid of the subject using the thumb or the index finger, infiltrated and touched the conjunctiva of lower eyelid with the sodium fluorescein test paper. The patient was required to look straight and blink 3–4 times. This allowed the examiner to observe the first dry spot (random position) in fluorescein on the tear film after the last blink by using the slit-lamp microscope.

#### CFS

One end of the sodium fluorescein test paper was infiltrated with aseptic saline. The examiners pulled the participants' eyelid down using the thumb or the index finger, and touched the lower eyelid conjunctiva of the subjects with the humid end. Then, the subjects were advised to lightly close the eyes to observe the staining of the corneal epithelium with the cobalt blue light of slit lamp. The examiner then divided the cornea into four quadrants when scoring (11). Each quadrant was divided into 0–3 points:

0: 0 dots
1: 1–5 dots
2: 6–30 dots
3: >30 dots

The four quadrant areas were added and recorded based on a standard of 0–12 points. Corneal epithelial staining was performed by the same examiner.

#### SIT

The subjects were oriented to maintain a calm and natural state. The examiner took a 35 mm long/5 mm wide standard test paper, folded the upper end of the test paper backward, and placed it gently in the middle and outside (1/3) of the conjunctive sac of the subject's lower eyelid. The rest of the palpebral fissure naturally hanged out of the eyelid. The whole process was completed following the principles of asepsis. The inspected patient was required to lightly close the eyes and look up, and could blink freely, and then lightly pulled down the eyelids and took out the test paper after 5 min, reading after another 2 min, and posted it for preservation.

### Statistical Methods

The result data were analyzed with SPSS 22.0 software. In this study, spearmen rank correlation was analyzed and correlation coefficients were calculated. *P* < 0.05 represents that the difference was statistically significant.

## Results

Twenty eight eyes of 28 subjects (25 females, 3 males; age range from 18 to 65 years old; averaged 46.3 ± 11.1 years old, Table 1) were examined. The mean SIT was 6.04 ± 4.64 mm/5 min (0–18 mm/5 min); the mean CFS was 3.07 ± 2.65 (0–10) and the mean BUT was 2.11 ± 1.97 s (0–9 s) (Table 2). The mean EF value was 0.52 ± 0.12 (0.26–0.75) in parotid glands and 0.45 ± 0.10 (0.30–0.67) in submandibular glands, respectively; the mean UI value was 9.33 ± 1.68 (6.03–13.20) in parotid glands and 9.92 ± 1.48 (7.08–12.60) in submandibular glands, respectively; the mean Ts (min) was 5.32 ± 3.01 (2.00–12.00) in parotid glands and 11.09 ± 7.40 (2.00–29.00 min) in submandibular glands, respectively (Table 3).

**Table 1:** Demographic information of the patients with Sjögren's syndrome (SS).

**The number of cases**	**Male (Cases)**	**Female (Cases)**	**Maximum age (years old)**	**Minimum age (years old)**	**Average age (years old)**
28	3	25	65	18	46.30 ± 11.1

**Table 2:** Summary of dry eye parameters in patients with Sjögren's syndrome (SS).

	**Mean**	**SD**	**Maximum**	**Minimum**
SIT (mm/5 min)	6.04	4.64	18.00	0.00
CFS	3.07	2.65	10.00	0.00
BUT (second)	2.11	1.97	9.00	0.00

*Tear film break-up time (TBUT), corneal fluorescein staining scoring (CFS), Schirmer's I test (SIT)*.

**Table 3:** Summary of quantitative parameters of SPECT examination in salivary glands.

	**Mean**	**SD**	**Maximum**	**Minimum**
**Parotid glands**				
EF	0.52	0.12	0.75	0.26
UI	9.33	1.68	13.20	6.03
T_s_ (min)	5.32	3.01	12.00	2.00
**Submandibular glands**				
EF	0.45	0.10	0.67	0.30
UI	9.92	1.48	12.60	7.08
T_s_ (min)	11.09	7.40	29.00	2.00

*Excretion fraction (EF). Uptake index (UI). The time needed to achieve the minimum counts after Vit C stimulation (T_s_)*.

**Figure 2:**
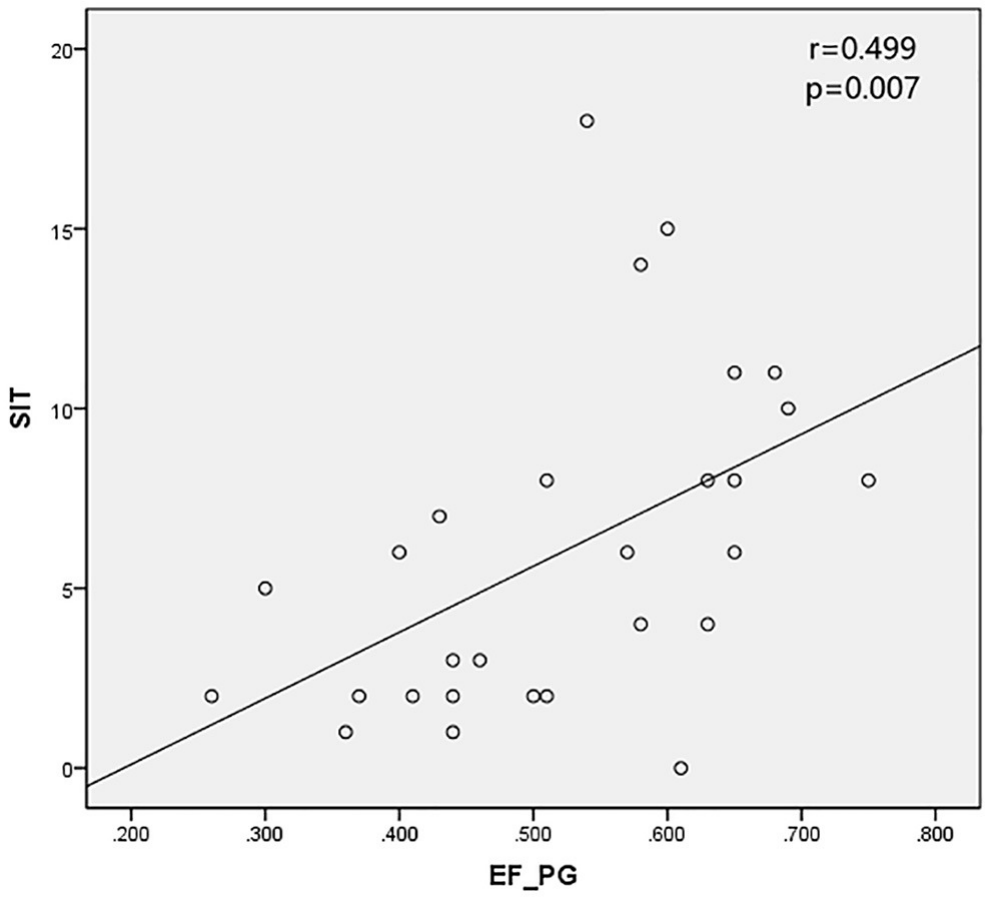
Excretion fraction in parotid glands (EF_PG) was positively correlated with SIT.

**Figure 3:**
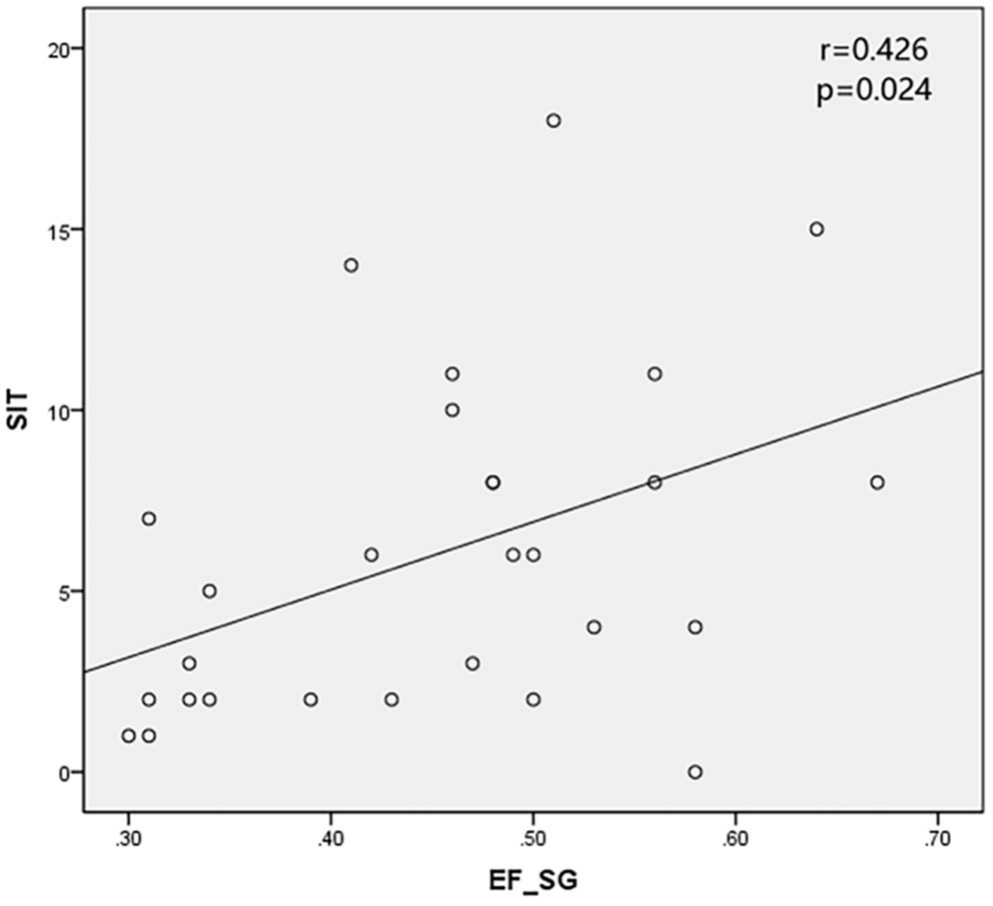
Excretion fraction in submandibular glands (EF_SG) was positively correlated with SIT.

The SPECT parameters were compared with TBUT, CFS, and SIT, accordingly. The excretion fraction was correlated with SIT in patients with SS (r = 0.499 and 0.426 in parotid glands and submandibular glands and *P* < 0.05). It was verified that UI and Ts do not have a significant correlation with CFS and TBUT (*P* > 0.05) (Table 4; Figures 2, 3).

**Table 4:** Correlation analysis of quantitative parameters of SPECT examination in salivary glands and dry eye tests.

**SPECT parameters**	**SIT**		**CFS**		**BUT**	
	**r**	***P*** **-value**	**r**	***P*** **-value**	**r**	***P*** **-value**
**Parotid glands**						
EF	0.499	0.007^*^	−0.145	0.462	0.272	0.162
UI	−0.028	0.887	0.164	0.403	0.258	0.186
T_s_ (min)	0.034	0.865	0.313	0.105	−0.087	0.660
**Submandibular glands**						
EF	0.426	0.024^*^	−0.298	0.124	0.164	0.405
UI	−0.091	0.644	0.173	0.378	0.123	0.533
T_s_ (min)	0.078	0.724	0.275	0.204	−0.114	0.606

*r, Pearson correlation coefficient*;

* *P < 0.05, the correlation was statistically significant*.

*Excretion fraction (EF). Uptake index (UI). The time needed to achieve the minimum counts after Vit C stimulation (T_s_)*.

## Discussion

The major characteristic of SS is the chronic autoimmune disorder of the exocrine glands with associated lymphocytic infiltrates of the affected glands (12). Sets of diagnostic criteria for SS have been proposed, the 2002 American-European Consensus Group (AECG) criteria (13) and 2012 American College of Rheumatology (ACR) criteria (14) were the most widely used in the medical community (15, 16) until 2016 ACR-EULAR criteria (3) was published. However, both the 2002 AECG criteria and 2012 ACR criteria have a high specificity (94.3% vs. 91.3%, respectively) but low sensitivity (61.6% vs. 62.3%, respectively) (17). We are constantly exploring new tests and methods for sensitive diagnose of SS.

The functional tests for the salivary glands were not included in the 2012 ACR criteria. The assessment of salivary glands was limited to the results of a minor salivary gland biopsy. The pathological features of minor salivary glands in SS patients were not exactly the same as those of major salivary glands (18). Furthermore, since the labial glands biopsy procedure is invasive, some patients may refuse it thus resulting in failure of the SS diagnosis. SPECT is currently considered a well-recognized test for the investigation of multiple diseases affecting salivary glands (19). In fact, radionuclide imaging of salivary gland yields only relatively low effective radiation doses compared with computed tomography (CT) for sialography (20). This dynamic, non-invasive, and quantitative detection assesses the functional state and degree of injury to the salivary glands.

Recently, SPECT has been refined to provide quantitative information on changes in salivary function (4). The use of computer-assisted time-activity curves allows quantification of salivary gland radioactivity turnover in a defined time period. However, which quantification parameter of salivary gland is best for assessing salivary gland dysfunction in SS patients remains inconclusive.

Delayed tracer uptake and secretion in SS patients is a progressive decrease in the exocrine function of the major salivary glands. This happens due to morphological changes caused by chronic immune-mediated inflammation. In order to improve the specificities of SPECT, we chose UI, T_s_, and EF as assessment parameters. The UI is defined as the tissue concentration of the tracer, measured by a scanner. It mainly assess the uptake function of the major salivary glands. T_s_ indicates how long the salivary glands secrete after acid stimulation. The EF value represents the secretory function of the salivary glands. In this study, decreased EF, UI and increased T_s_ are considered as a result of a progressive reduction in the exocrine function of the major salivary glands.

Schirmer's I test was developed nearly 100 years ago and the method has been widely used for evaluation of tear production. It is a well-standardized test that can be used to estimate stimulated reflex aqueous secretion (10) by observing the degree on the folded strip. The secretion disorder caused by the exocrine gland damage is the main pathological process of SS patients (12). The reduction of lacrimal gland secretion in SS patients leads to the decrease of SIT. The correlation between SIT and EF in this study may be due to the consistent degree of damage to the exocrine glands in SS. There is no significant correlation between SIT and T_s_ maybe because of the insensitivity of T_s_ as an indicator of salivary gland secretion. UI, as an indicator of uptake function, was not correlated with other indicators in this trial, possibly because the uptake function of the glands in SS patients was not much damaged. It's reported there's no difference in UI value between SS patients and non-SS patients (5). Studies involving more subjects are needed to confirm this conclusion.

Some other widely used diagnostic methods for dry eye are BUT and CFS. The BUT is defined as the time interval between a blink and the first occurrence of gaps or breaks in the tear film and it mainly assesses the tear film stability. The tear film lipid layer is derived from the meibum secreted from the lid margins and is spread onto the tear film after each blink. It plays an important role in stabilizing the tear film (21). Thus, the BUT has no significant correlation with the UI, T_s_, and EF in our results.

Corneal and conjunctival staining are considered informative markers of severity degree in severe DED cases, whereas mild/moderate ocular surface staining is less associated with disease severity (22). Fluorescein sodium staining can be observed when the corneal epithelium is damaged. The classical theory is that the dye, which is dissolved in saline and is thus hydrophilic, is transported across altered tight junctions in diseased patients (23). A punctate cornea stain is actually a small lump of dye where cells are missing. Thus, CFS mainly reflects the integrity of the corneal epithelium rather than the secretion of tears. Hence, it is not significantly correlated with UI, Ts, and EF in our results.

SPECT has unique diagnostic advantages for patients with SS since it provides a non-invasive, dynamic, and quantitative measure of exocrine glands function. The lacrimal gland is a small part of the orbital structures, which makes it difficult to obtain biopsy materials. With the successful clinical application of positron emission tomography (PET)/CT in recent years, the next step is to produce a higher resolution SPECT/CT hybrid scanner (24). Then, the dynamic detection of lacrimal gland function will be possible.

## Conclusion

SPECT can help examiners and doctors to better understand the decreased secretion velocity in the submandibular and parotid glands. The EF was positively correlated with SIT in patients with SS, and could reflex the dysfunction of salivary glands in SS patients. EF may be a valuable parameter for the diagnosis of SS patients with lacrimal gland secretion dysfunction.

## Data Availability Statement

The raw data supporting the conclusions of this article will be made available by the authors, without undue reservation.

## Ethics Statement

The studies involving human participants were reviewed and approved by the Ethics Committee of Tongji Hospital, Tongji Medical College, Huazhong University of Science and Technology. The patients/participants provided their written informed consent to participate in this study. Written informed consent was obtained from the individual(s) for the publication of any potentially identifiable images or data included in this article.

## Author Contributions

XH: acquisition of data, conception and design of study, analysis and/or interpretation of data, and drafting the manuscript. GL and JC: revising the manuscript critically for important intellectual content. All authors contributed to manuscript revision, read, and approved the submitted version.

## Funding

This work was supported by the National Natural Science Foundation of China (Nos. 81470606, 81570819, and 82070936), the Nature Science Foundation of Hubei Province (No. 2014CFB973), Hubei Province health and family planning scientific research project (WJ2017M073), Top ten translational medical research projects from Tongji Hospital (No. 2016ZHYX20), and Training project of young medical pioneers in Wuhan city (No. 2015whzqnyxggrc10).

## Conflict of Interest

The authors declare that the research was conducted in the absence of any commercial or financial relationships that could be construed as a potential conflict of interest.

## Publisher's Note

All claims expressed in this article are solely those of the authors and do not necessarily represent those of their affiliated organizations, or those of the publisher, the editors and the reviewers. Any product that may be evaluated in this article, or claim that may be made by its manufacturer, is not guaranteed or endorsed by the publisher.
